# Variability in stroke motor outcome is explained by structural and functional integrity of the motor system

**DOI:** 10.1038/s41598-018-27541-8

**Published:** 2018-06-21

**Authors:** Timothy K. Lam, Malcolm A. Binns, Kie Honjo, Deirdre R. Dawson, Bernhard Ross, Donald T. Stuss, Sandra E. Black, J. Jean Chen, Takako Fujioka, Joyce L. Chen

**Affiliations:** 1 Heart and Stroke Foundation Canadian Partnership for Stroke Recovery, Toronto, ON Canada; 2 Hurvitz Brain Sciences Research Program, Sunnybrook Research Institute, Toronto, ON Canada; 3 Rehabilitation Sciences Institute, University of Toronto, Toronto, ON Canada; 4 Rotman Research Institute, Baycrest Centre, Toronto, ON Canada; 5 Dalla Lana School of Public Health, University of Toronto, Toronto, ON Canada; 6 Department of Occupational Science and Occupational Therapy, University of Toronto, Toronto, ON Canada; 7 Department of Medical Biophysics, University of Toronto, Toronto, ON Canada; 8 Department of Psychology, University of Toronto, Toronto, ON Canada; 9 Department of Medicine (Neurology), University of Toronto, Toronto, ON Canada; 10 Center for Computer Research in Music and Acoustics, Department of Music, Stanford Neurosciences Institute, Stanford University, Stanford, CA USA; 11 Department of Physical Therapy, University of Toronto, Toronto, ON Canada

## Abstract

Biomarkers that represent the structural and functional integrity of the motor system enable us to better assess motor outcome post-stroke. The degree of overlap between the stroke lesion and corticospinal tract (CST Injury) is a measure of the structural integrity of the motor system, whereas the left-to-right motor cortex resting state connectivity (LM1-RM1 rs-connectivity) is a measure of its functional integrity. CST Injury and LM1-RM1 rs-connectivity each individually correlate with motor outcome post-stroke, but less is understood about the relationship between these biomarkers. Thus, this study investigates the relationship between CST Injury and LM1-RM1 rs-connectivity, individually and together, with motor outcome. Twenty-seven participants with upper limb motor deficits post-stroke completed motor assessments and underwent MRI at one time point. CST Injury and LM1-RM1 rs-connectivity were derived from T1-weighted and resting state functional MRI scans, respectively. We performed hierarchical multiple regression analyses to determine the contribution of each biomarker in explaining motor outcome. The interaction between CST Injury and LM1-RM1 rs-connectivity does not significantly contribute to the variability in motor outcome. However, inclusion of both CST Injury and LM1-RM1 rs-connectivity explains more variability in motor outcome, than either alone. We suggest both biomarkers provide distinct information about an individual’s motor outcome.

## Introduction

A biomarker is “an indicator of disease state that can be used clinically as a measure reflecting underlying molecular/cellular processes that may be difficult to measure directly in humans”^1^. Neurological biomarkers derived from neuroimaging data and non-invasive brain stimulation approaches provide important information about a patient’s potential for neurobiological recovery^2–4^. Furthermore, the combination of multiple biomarkers together can lead to more robust prediction models of motor recovery after stroke^3,5^. Thus, the global objective of the present research is to evaluate the role of two neuroimaging biomarkers on explaining variability in motor outcome after stroke, the integrity of the corticospinal tract (CST) and motor resting state connectivity. These biomarkers have each been the focus of several studies^6–11^, however little is known about how they interact or add together to explain the variability in motor outcome. Here, we refer to motor outcome as the clinical status of an individual at one time point after their stroke.

The CST connects the primary motor cortex (M1) with the spinal cord, and is the major motor output pathway in humans. The structural integrity of the motor system post-stroke can be quantified by the amount of injury to the CST, defined as the percent of overlap between the stroke lesion and CST (CST Injury)^3,12^. Individuals with stroke who have greater CST Injury have worse motor outcome than those with lower CST Injury^6–8,13^. Damage to motor brain regions including M1 also lead to deficits^14,15^. The functional integrity of the motor system can be assessed using resting state functional magnetic resonance imaging (rs-fMRI). Rs-fMRI measures the spontaneous fluctuations in neural activity (i.e., blood oxygen level dependent (BOLD) signal) when the brain is not engaged in any task^16^. In healthy individuals, the BOLD signal between left and right M1 are temporally coupled, and are thus considered ‘connected’^17^. Individuals with stroke with damage to their motor system show disrupted resting state connectivity between the left and right M1 (LM1-RM1 rs-connectivity) relative to healthy controls^10^ and those with higher LM1-RM1 rs-connectivity have better motor outcome than those with lower LM1-RM1 rs-connectivity (see^18^ for a review). Taken together, CST Injury and LM1-RM1 rs-connectivity are biomarkers that represent the structural and functional integrity of the motor system, respectively, and explain variance in motor outcome post-stroke.

But CST Injury and LM1-RM1 rs-connectivity also interact with each other. In the early sub-acute stage (7 days-3 months) post-stroke^1^, CST Injury and LM1-RM1 rs-connectivity negatively correlate with each other^13^. Moreover, the relationship between LM1-RM1 rs-connectivity and motor outcome is modulated by CST Injury whereby LM1-RM1 rs-connectivity correlates with motor outcome only at low levels of CST Injury^13^. It is unknown, however, whether the interaction between these two biomarkers persists in the late sub-acute (3–6 months) and chronic (>6 months) stage. During this time, the brain’s endogenous plasticity and hence spontaneous neural repair has tapered. Alternatively, if there is no interaction, do both biomarkers together explain more variability in motor outcome than either biomarker alone. Therefore, the main objective of the present study was to determine whether the relationship between CST Injury and LM1-RM1 rs-connectivity in the chronic stage post-stroke is interactive, based on prior findings in individuals with early sub-acute stroke^13^, or additive. We used a hierarchical regression analysis to test two models: 1) an interactive model that included an interaction term of CST Injury and LM1-RM1 rs-connectivity, and 2) an additive model of CST Injury and LM1-RM1 rs-connectivity without an interaction term. We also aimed to confirm prior findings that these biomarkers contribute individually in explaining the variability in motor outcome. If there is an interaction, we hypothesize that there is a threshold of CST Injury, from which LM1-RM1 rs-connectivity will explain the variability in motor outcome. If there is no interaction and these biomarkers are additive, we hypothesize that CST Injury and LM1-RM1 rs-connectivity together explain more variability in motor outcome than one biomarker alone. Lastly, we hypothesize that individuals with stroke who have less CST Injury and greater LM1-RM1 rs-connectivity will have better motor outcome.

## Results

### Behaviour

27 participants with unilateral upper limb motor deficits in the chronic stage of stroke completed motor assessments and magnetic resonance imaging (MRI). Two participants were excluded from the analyses (described below) since their stroke lesion completely overlapped the arm-hand region of their M1, which is a region of interest in our study. In total, we present and analyze the data for 25 participants. Participants completed the Chedoke-McMaster Stroke Assessment (CMSA) Stage of Arm (CMSA-Arm) and Stage of Hand (CMSA-Hand) to assess motor impairment in the arm and hand, respectively. The CMSA has good validity with the Fugl-Meyer Assessment^19^. To obtain a single measure of motor impairment (CMSA-Motor) for the upper limb, we summed the CMSA-Arm and CMSA-Hand scores. The CMSA-Motor score ranges from 2 (flaccid paralysis in the arm and hand) to 14 (normal movement in the arm and hand). Participants also completed the Action Research Arm Test (ARAT) as a measure of motor function^20^. Table 1 (group data) and Supplementary Table S1 (individual data) summarize participant demographics and performance on the motor assessments.

**Table 1:** Participant demographics and performance on motor assessments.

***Demographics*** (N = 25)	
Age, years	61.9 ± 10.2 (41–79)
Sex	
Male	17 (68%)
Female	8 (34%)
Time since stroke, months	60.0 ± 69.6 (7–305)
Education, years	15.6 ± 2.9 (10–22)
Lesion location	
Left hemisphere	11 (44%)
Right hemisphere	14 (56%)
Dominant hand affected*	
Yes	9 (38%)
No	15 (62%)
***Neurological Measures***	
Injury to the corticospinal tract, percent	46.3 ± 28.1 (1.6–100)
Resting state connectivity between left and right primary motor cortex, *r*-value	0.45 ± 0.26 (0.17–0.79)
***Motor Assessments***	
Chedoke-McMaster Stroke Assessment Impairment Inventory Total Motor Impairment Score	6.4 ± 1.8 (4–10)
Action Research Arm Test	27.2 ± 21.4 (0–57)

Data are presented as mean ± SD (range of variable) for continuous variables, and number of participants (percent of sample) for categorical variables. For participants with bilateral lesions (s05, s15, s21), lesion hemisphere was determined according to clinical presentation of the upper limb deficit. *Handedness was not collected for the first participant.

### MRI

**Figure 1:**
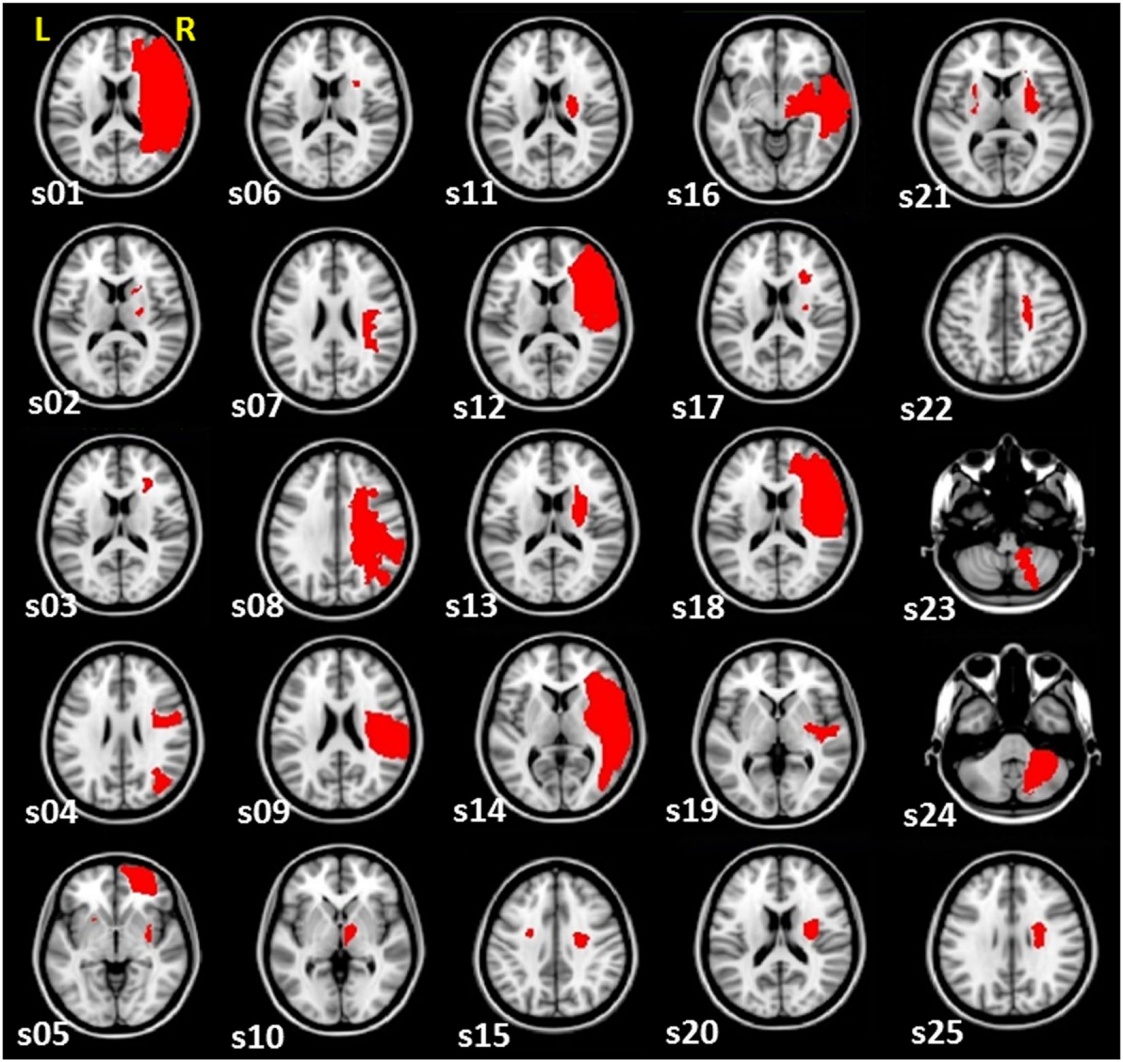
Lesion masks of participants. Lesion tracings for each participant are displayed (in red) on the right hemisphere and superimposed on a Montreal Neurological Institute (MNI ICBM) 2 mm template. The transverse slice of the lesion with the largest cross-sectional area is displayed. (“s” indicates subject; “L” indicates left; “R” indicates right).

**Figure 2:**
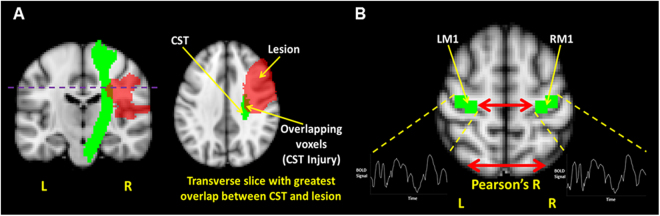
Structural and functional biomarkers. A schematic that depicts the structural and functional integrity measures of the motor system studied in the present study. (**A**) Injury to the corticospinal tract (CST Injury) is determined using the transverse slice (purple dotted line) of the corticospinal tract (CST) template (green) that has the maximum overlap with the lesion mask (red). The purple dotted line depicts the transverse slice of the CST in which CST Injury is determined, given that the overlap between the CST and lesion is greatest at this slice. (**B**) The resting state connectivity between left and right primary motor cortex (LM1-RM1 rs-connectivity) is determined by the Pearson’s *r*-value between the mean BOLD time series of the left and right primary motor cortex. Images are overlaid on a standard Montreal Neurological Institute (MNI ICBM) 2 mm template. “L” indicates left and “R” indicates right.

To determine CST Injury and LM1-RM1 rs-connectivity in each participant, we obtained structural T1-weighted and rs-fMRI scans, respectively. Lesion tracings for each participant are depicted in Fig. 1. Based on the method by Pineiro *et al*.^6^, we calculated CST Injury on the transverse slice of the CST with the greatest overlap with the lesion [Fig. 2A]. To calculate LM1-RM1 rs-connectivity, we created seed masks for LM1 and RM1 derived from arm/elbow and hand/finger fMRI paradigms (Supplementary Table S2). We then extracted the mean time series of each seed from the rs-fMRI data, and computed the Pearson’s correlation (*r*-value) for a measure of LM1-RM1 rs-connectivity in each participant [Fig. 2B]. Two participants (s01 and s08) had lesions that partially overlap the RM1 seed. The percent of the RM1 seed that overlapped the lesion for s01 and s08 was 34% and 10%, respectively. We included these two participants in the analyses since the perilesional tissue in the right M1 region appears mostly intact.

### Statistical analyses

#### Hierarchical regression analysis to explain variability in the CMSA-Motor and ARAT scores

We derived four multiple regression models to explain the variability in the CMSA-Motor score. We first developed an interaction model with three terms (CST Injury, LM1-RM1 rs-connectivity, and their interaction) to explain the variability in CMSA-score. We then developed an additive model with two terms (CST Injury and LM1-RM1 rs-connectivity). Lastly, we developed two simple regression models, each with one term, to explain the variability in CMSA-score; the first simple regression model included CST Injury and the second simple regression model included LM1-RM1 rs-connectivity. This statistical procedure was repeated to explain the variability in the ARAT score.

To address our study objective, we determined the R^2^ change (ΔR^2^) value to compare whether the different models were significantly different from each other. The ΔR^2^ value represents the difference in the amount of explained variability between two models. We derived the ΔR^2^ value to compare between the interaction and additive models, and between the additive and simple regression models.

**Figure 3:**
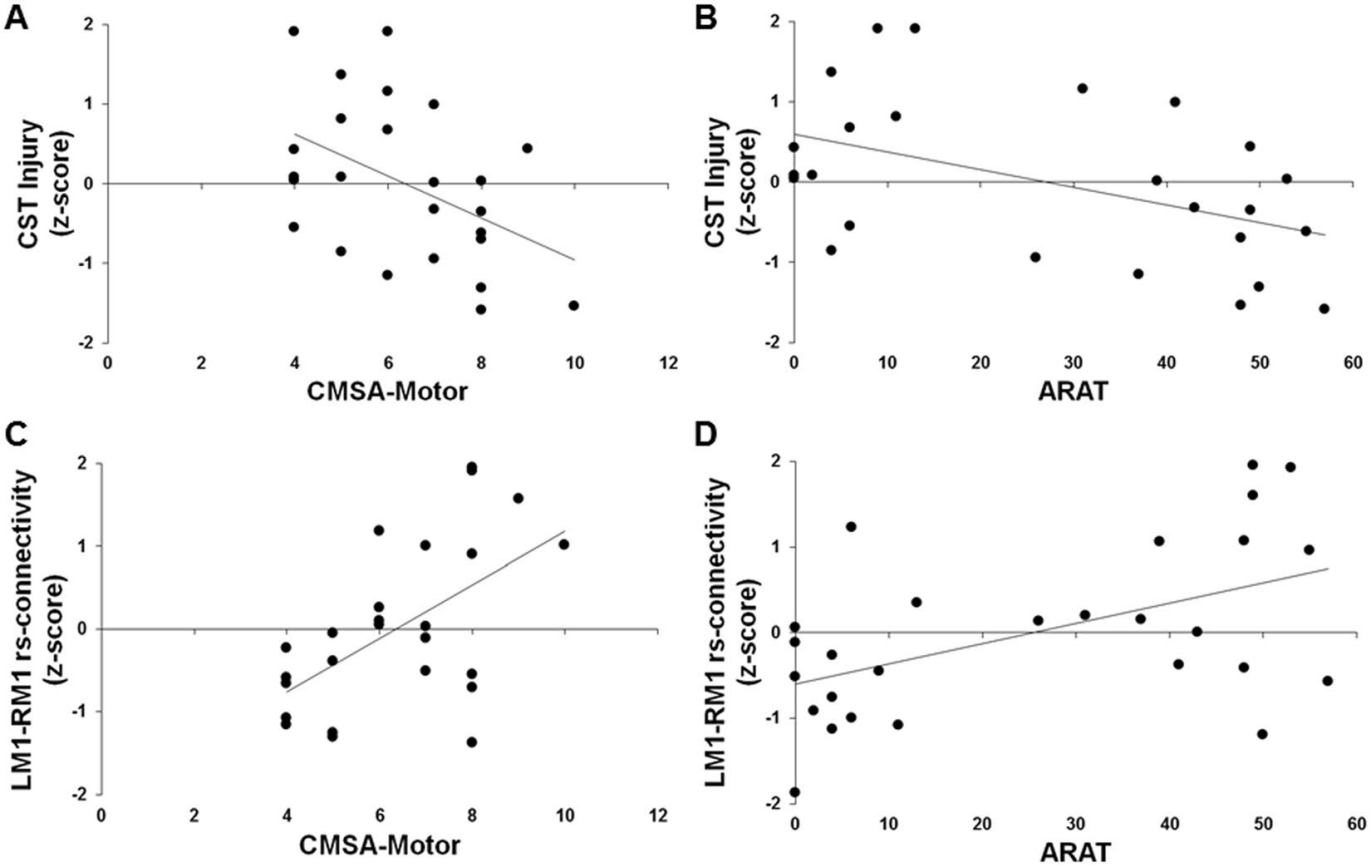
Relationship between structural and functional biomarkers and motor outcome. Scatterplots of biomarkers and motor assessment scores. Injury to the corticospinal tract (CST Injury) accounts for 23% variance in (**A**) the Chedoke-McMaster Stroke Assessment: Impairment Inventory Total Motor Impairment (CMSA-Motor) score, and 24% variance in (**B**) the Action Research Arm Test (ARAT) score. Resting state connectivity between left and right primary motor cortex (LM1-RM1 rs-connectivity) accounts for 34% variance in (**C**) the CMSA-Motor score, and 22% variance in (**D**) the ARAT score.

Table 2 summarizes the interaction, additive, and simple regression models to explain variability in the CMSA-Motor and ARAT scores. The residuals of the CMSA-Motor and ARAT scores in each model were approximately normally distributed. The interaction models were not significantly different from the additive models; the interaction term accounted for a non-significant additional 0.2% variance in both the CMSA-Motor score (ΔR^2^ = −0.002, *p* = 0.75) and ARAT score (ΔR^2^ = −0.002, *p* = 0.81). The additive model was significantly different from simple regression model 1 with only CST Injury; the inclusion of LM1-RM1 rs-connectivity accounted for an additional 34% of the variance in the CMSA-Motor score (ΔR^2^ = −0.34, *p* < 0.001) and 22% of the variance in the ARAT score (ΔR^2^ = −0.22, *p* = 0.008). The additive model was also significantly different from simple regression model 2 with only LM1-RM1 rs-connectivity; the inclusion of CST Injury significantly accounted for an additional 23% of the variance in the CMSA-Motor score (ΔR^2^ = −0.23, *p* = 0.003) and 24% of the variance in the ARAT score (ΔR^2^ = −0.24, *p* = 0.006). Thus, both CST Injury and LM1-RM1 rs-connectivity significantly contribute to explaining the variability in CMSA-Motor and ARAT scores [Fig. 3].

**Table 2:** Hierarchical Multiple Regression for CMSA-Motor and ARAT.

	R^2^	Adjusted R^2^	*p*-value	β	*p*-value	ΔR^2^	*p*-value
***Chedoke-McMaster Stroke Assessment Impairment Inventory Total Motor Impairment (CMSA-Motor)***							
Interaction Model	0.55	0.49	0.001			—	—
CST Injury				−0.48	0.004		
LM1-RM1 rs-connectivity				0.60	0.001		
Interaction				0.05	0.75		
Additive Model	0.55	0.51	<0.001			−0.002^#^	0.75
CST Injury				−0.48	0.003		
LM1-RM1 rs-connectivity				0.58	<0.001		
Simple Regression 1	0.21	0.18	0.02			−0.34^^^	<0.001
CST Injury				−0.46	0.02		
Simple Regression 2	0.32	0.29	0.003			−0.23^^^	0.003
LM1-RM1 rs-connectivity				0.57	0.003		
***Action Research Arm Test (ARAT)***							
Interaction Model	0.44	0.36	0.006			—	—
CST Injury				−0.48	0.008		
LM1-RM1 rs-connectivity				0.48	0.01		
Interaction				0.04	0.81		
Additive Model	0.44	0.39	0.002			−0.002^#^	0.81
CST Injury				−0.49	0.006		
LM1-RM1 rs-connectivity				0.47	0.008		
Simple Regression 1	0.22	0.19	0.02			−0.22^^^	0.008
CST Injury				−0.47	0.02		
Simple Regression 2	0.20	0.17	0.02			−0.24^^^	0.006
LM1-RM1 rs-connectivity				0.45	0.02		

R^2^, adjusted R^2^, β-values, ΔR^2^ values, and the associated significance (*p*-values) for the hierarchical multiple regression models to explain variability in performance on motor assessments. Hash (^#^) represents the ΔR^2^ value from the comparison between the interaction model and additive model. Caret (^^^) represents the ΔR^2^ value from the comparison between the additive model and simple regression model. Model comparisons are considered significant at *p* < 0.05.

#### Supplementary hierarchical regression analyses

The inclusion of covariates (i.e., age, sex, time since stroke, education, and dominant hand affected) did not significantly influence the results from the regression models [Supplementary Tables S3–S7], and hence these covariates were not included in the final models presented here. The results from the interactive, additive, and simple regression models remain the same when excluding participants with bilateral (s05, s15, s21) or cerebellar (s23, s24) lesions [Supplementary Tables S8,S9]. The pattern of findings also remains the same when removing the two participants with partial overlap between the RM1 seed and stroke lesion [Supplementary Table S10].

#### Correlation between CST Injury and LM1-RM1 rs-connectivity

**Figure 4:**
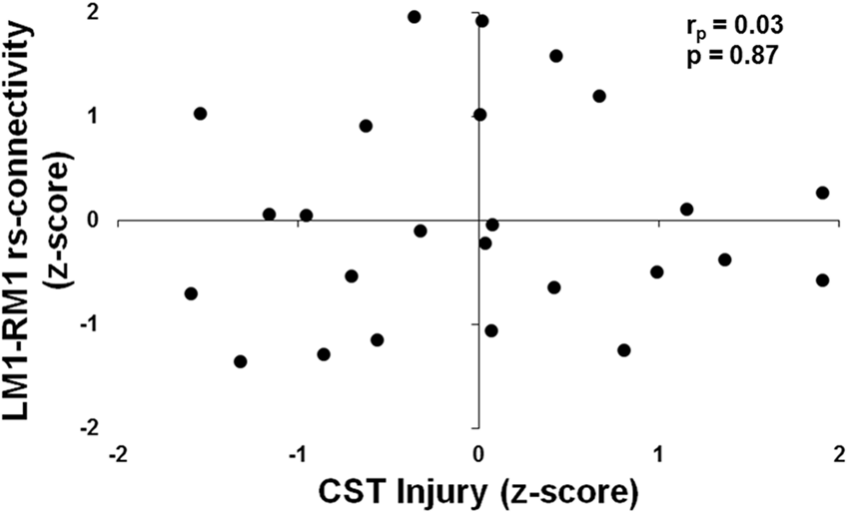
Relationship between structural and functional biomarkers. Scatterplot between injury to the corticospinal tract (CST Injury) and resting state connectivity between left and right primary motor cortex (LM1-RM1 rs-connectivity). The linear relationship between these two biomarkers was not significant.

Given that the interaction term did not significantly contribute to explaining the variability in motor outcome, we performed a Pearson’s correlation between CST Injury and LM1-RM1 rs-connectivity to determine the relationship between these biomarkers. No significant relationship was detected between CST Injury and LM1-RM1 rs-connectivity (r_p_(23) = 0.03, *p* = 0.87) [Fig. 4]. The relationship between CST Injury and LM1-RM1 rs-connectivity remained non-significant after excluding participants with bilateral (r_p_(20) = 0.04, *p* = 0.85) or cerebellar (r_p_(21) = 0.03, *p* = 0.88) lesions.

## Discussion

We examined the relationship between CST Injury and LM1-RM1 rs-connectivity as biomarkers of the motor system to explain the variability in motor outcome in individuals with chronic stroke. We replicate prior work whereby CST Injury and LM1-RM1 rs-connectivity individually explain the variability in motor outcome. The interaction between CST Injury and LM1-RM1 rs-connectivity was not found to significantly account for variability in motor outcome. However, the novel finding is that the inclusion of both CST Injury and LM1-RM1 rs-connectivity together significantly explains the variability in motor outcome more than either biomarker alone. Our results suggest that both CST Injury and LM1-RM1 rs-connectivity are biomarkers that provide distinct information on motor outcome in individuals with chronic stroke.

The CST conveys signals from motor cortex to the spinal cord, and direct damage to it leads to motor deficits that include paresis and paralysis. We replicate prior work showing that participants with stroke who have greater CST Injury have more impairment (i.e., lower CMSA-Motor score) and worse motor function (i.e., lower ARAT score) than those with less CST Injury^6,7,13,21^. It is thought that CST Injury primarily assesses the degree of motor impairment^7,21–23^. The resolution of impairment is linked with the brain’s true or spontaneous recovery in response to the stroke^24^. However, deficits in motor impairment and function are related, which may be a reason why our findings and those of others show a relationship between CST Injury and motor function. An individual with motor impairment (e.g. weakness in the elbow extensors) may be unable to perform some motor functions (e.g. reach for a cup of coffee), unless motor behaviours are adapted using compensatory strategies (e.g. lean forward with trunk to reach for cup).

We show that participants with stroke who have higher LM1-RM1 rs-connectivity have less impairment than those with lower LM1-RM1 rs-connectivity. This result replicates prior work showing LM1-RM1 rs-connectivity correlates with motor impairment and function post-stroke^9–11^. Resting state connectivity, the coherent spontaneous fluctuations in the BOLD signal, reflects an intrinsic property of functional brain organization^25^. Patterns of LM1-RM1 connectivity at rest are similar to those elicited during motor task performance^17^, and contribute to variability in motor behaviour^26^. However, the specific behavioural construct represented by LM1-RM1 rs-connectivity in stroke is not well understood. It is unclear whether connectivity indices reflect the state of motor impairment, functioning, or both. That is, do connectivity changes reflect a mitigation of impairment that corresponds to true recovery, or, enhanced motor functioning from a combination of true recovery and compensatory strategies? There is a similar lack of agreement over the interpretation of task-based fMRI findings in people with stroke^27,28^ since brain activation patterns may represent true recovery, behavioural compensation, or both^27^. Future work may elucidate the differential contributions of recovery and compensatory processes that underlie motor rs-connectivity signals.

We did not detect a significant interaction between CST Injury and LM1-RM1 rs-connectivity in the regression models for motor outcome. The interaction term in our model explained a non-significant additional 2% of the variance in the CMSA-Motor score, and 0.2% of the variance in the ARAT score. Furthermore, the correlation between CST Injury and LM1-RM1 rs-connectivity was not significant. As described previously, we suggest that CST Injury and LM1-RM1 rs-connectivity may represent distinct measures of motor outcome.

Our findings are in contrast to two previous studies. Carter *et al*.^13^ found that in individuals with sub-acute stroke CST Injury negatively correlates with LM1-RM1 rs-connectivity; more injury is associated with lower connectivity. Carter *et al*.^13^ also found that for people with less than 10% damage to the CST better motor outcome is associated with higher motor connectivity. In contrast, Liu *et al*.^29^ used fractional anisotropy (FA) to assess CST integrity, and found the opposite of Carter *et al*.^13^. People with higher FA, which could be interpreted as less injury to the CST, have lower LM1-RM1 rs-connectivity. Liu *et al*.^29^ suggest that LM1-RM1 rs-connectivity may reflect a compensatory response. Individuals with higher FA (less CST injury) may not need to rely heavily on recruitment of compensatory motor brain regions to facilitate their motor recovery, in contrast to people who have lower FA (more CST injury).

Thus, there are three different findings regarding the nature of the relationship between CST Injury and LM1-RM1 rs-connectivity: no relationship (findings of the present study), a negative relationship (Carter *et al*.^13^), and a positive relationship (Liu *et al*.^29^). Upon closer inspection, there are several reasons why findings do not appear to converge. First, we and Liu *et al*.^29^ studied individuals with chronic stroke, greater than 6 months, whereas Carter *et al*.^13^ studied individuals in the early subacute stage, less than 4 weeks, post-stroke. A major difference between these stages is that spontaneous recovery dominates in the latter, but not in the former^30,31^. Spontaneous recovery appears fixed, as individuals with stroke recover approximately 70% of their maximum recovery potential by the end of the subacute stage, and recovery potential is not influenced by therapy dose^21,23,32–35^. Spontaneous recovery has an overarching influence on motor outcome in the sub-acute stage such that both CST Injury and LM1-RM1 rs-connectivity may be biomarkers reflective of motor impairment and thus correlate with each other. However, spontaneous recovery no longer dominates in the chronic stage, and people with stroke learn compensatory strategies based on their residual motor ability, which likely determines the extent of motor recovery^24^. In the chronic stage, compensation may be reflected in LM1-RM1 rs-connectivity, but not CST Injury, as the latter measures the degree of structural damage that may be relatively fixed from stroke onset. Therefore, these biomarkers, when evaluated in the chronic stage post-stroke, may not correlate with each other because each reflects different aspects of motor behaviour. This interpretation is supported by findings from other studies in individuals with chronic stroke that also did not find a structure-function relationship^11,36^.

Although both Liu *et al*.^29^ and our study examined individuals with chronic stroke, the relationship between CST damage and LM1-RM1 rs-connectivity was observed in the former but not in the latter. This discrepancy may in part, be due to the different methods used to calculate CST damage. Liu *et al*. used FA, whereas we used the amount of overlap between the lesion and CST. FA is a non-specific measure of CST integrity since FA values can be influenced by different factors, such as crossing fibers and axon diameter^37^. Furthermore, Liu *et al*. studied individuals with subcortical strokes who also had good motor outcome (>90/100 on the Fugl-Meyer Assessment). In contrast, participants in our study have variable lesion locations and poor-to-moderate motor outcome with CMSA-Arm and CMSA-Hand stages that range from 2–5 (see Supplementary Table S1).

Taken together, each of the studies discussed here (Carter *et al*.^13^, Liu *et al*.^29^, and ours) have tested people in different stages post-stroke with different levels of motor outcomes, which unsurprisingly have yielded different findings. This highlights the need for larger scale studies that include the testing of individuals with stroke spanning all stages of chronicity and severity. Only then, might we be able to parse and understand how brain structure-function relationships manifest post-stroke.

Our novel finding is that both CST Injury and LM1-RM1 rs-connectivity together significantly contribute to the overall regression model, explaining more variability in motor impairment and function than one biomarker alone. The additive model explains 51% and 39% of the CMSA-Motor and ARAT scores, respectively. The simple regression model 1 with CST Injury explained 18% of the variance in the CMSA-Motor score, and 19% of the variance in the ARAT score. Comparison between the additive and simple regression 1 models showed that the inclusion of LM1-RM1 rs-connectivity in the additive model contributed an additional 34% and 22% of the variance in the CMSA-Motor and ARAT scores, respectively. The simple regression model 2 with LM1-RM1 rs-connectivity explained 29% of the variance in the CMSA-Motor score, and 17% of the variance in the ARAT score. Comparison between the additive and simple regression 2 models showed that the inclusion of CST Injury in the additive model contributed an additional 23% and 24% of the variance in the CMSA-Motor and ARAT scores, respectively. Collectively, these results suggest that one biomarker does not completely describe movement after stroke since the additive model explains more variance in motor performance than the simple regression models. As we previously posit, CST Injury and LM1-RM1 rs-connectivity may each reflect distinct information about the state of the motor system in individuals with chronic stroke. CST Injury is a structural biomarker of impairment, whereas LM1-RM1 rs-connectivity is a functional biomarker of motor behaviour. Our data appear to show that a combination of biomarkers may allow for better characterization of chronic stroke motor outcome.

In fact, consensus-based core recommendations from an international Stroke Recovery and Rehabilitation Roundtable^2^ highlight the importance of incorporating multiple biomarkers, which when combined together, have strong predictive value. Other studies have evaluated different combinations of structural and functional biomarkers that explain variability in motor outcome at one time point^36,38,39^. Wu *et al*.^36^ showed that EEG-based motor connectivity measures along with amount of CST injury together explained 93% of variance in motor impairment in twelve individuals with chronic stroke. Volz *et al*.^38^ showed that the active motor threshold (assessed by transcranial magnetic stimulation (TMS)), interhemispheric connectivity (assessed by dynamic causal modelling), and CST injury, together accounted for more than 80% of variance in motor impairment, in twelve individuals with chronic stroke. A novel algorithm demonstrated that for people with stroke without a motor evoked potential, FA of the CST can characterize the extent of upper limb recovery^39,40^. While CST Injury is a major determinant of motor impairment and is ‘ready to be used in clinical trials’^2^, the role of other motor regions including M1 should not be discounted^5,41^. Furthermore, priority research areas include the need to systematically evaluate the utility of rs-connectivity measures^2^.

There are a few limitations to our study. First, the less-affected limb may also be impaired post-stroke^42^. While we did not observe any deficits in our participants, this was not formally assessed. Modeling motor impairment and function in both upper limbs may allow for a more precise characterization of structural and functional brain changes. Second, the CST template, derived from the Johns Hopkins University (JHU) white matter atlas, may also include fibers from other M1 regions, such as the lower limb and face. Thus, the CST Injury values may not solely represent upper limb deficits since these values may be influenced by motor impairment in these other body regions. Third, we also only examined data from one time point and thus cannot draw conclusions about whether CST Injury and LM1-RM1 rs-connectivity predict motor recovery. Future studies may employ a longitudinal design to inform how these biomarkers predict motor recovery, especially at the individual level. Furthermore, future efforts may attempt to pinpoint which structural and functional biomarkers are most sensitive in predicting outcomes, and understand the behavioural constructs represented by these biomarkers across the different time-points post-stroke. In this vein, the use of kinematic information can more sensitively characterize an individual’s motor status^43^, which may lead to a deeper understanding for how behaviour maps onto brain structure and function. For these biomarkers to be applied in a clinical context, we must also consider the feasibility of data collection (e.g. are MRI scans clinically available). Once key biomarker candidates are identified, collaborative studies can pool together a large sample of participants to validate their utility and reliability^44^.

We show that CST Injury and LM1-RM1 rs-connectivity each contribute to explaining variability in motor outcome. However, a non-significant interaction between these biomarkers suggests that the relationship between CST Injury and LM1-RM1 rs-connectivity may in fact be additive. Thus, CST Injury and LM1-RM1 rs-connectivity may provide distinct information on the motor system, thereby allowing one to better understand and predict motor ability after stroke.

## Methods

### Participants

Twenty-seven participants with chronic stroke provided informed written consent for a clinical trial (NCT01721668) approved by the Baycrest Research Ethics Board. All methods were performed in accordance with approved institutional guidelines and regulations. The clinical trial examined the effectiveness of an arm and hand intervention delivered over ten weeks to individuals with upper limb deficits during the chronic stage of stroke. In the present study we excluded two participants since their stroke lesion completely overlapped the arm-hand region of their M1, which is a region of interest in our study. In total, we present and analyze the data for 25 participants. We analyzed a subset of data collected at baseline (pre-intervention stage) that are unrelated to the objectives of the clinical trial. Thus, only details relevant to the current study are reported here. Details of the clinical trial will be reported in future publications.

Inclusion criteria were: first-time stroke at least six-months from onset, with a clinical presentation of unilateral upper limb deficit, English-speaking, near-normal hearing verified by clinical audiometry (<40 dB 250–2000 Hz), and residual motor impairment. Motor impairment was assessed for the upper limb affected by the stroke and characterized by at least a stage 2 on the CMSA-Arm and CMSA-Hand^19^. Individuals at stage 2 were also required to complete at least one task in stage 3 of the CMSA. Exclusion criteria were: moderate to severe apraxia and/or aphasia, sensory loss, clinically significant spatial neglect, dementia, psychiatric disorders, severe pain and/or fatigue. Participants were also excluded if they were concurrently participating in another clinical intervention trial during the study period, had formal music training for more than 2 years within the past 10 years, or for more than 10 years in total, and if they were significantly depressed (Center for Epidemiological Studies-Depression scale, with higher scores indicating more depressive symptoms)^45^. Any participant on antidepressants must have been on a stable dosage for at least 3 months with no change during the study period.

### Assessments

Participants underwent the following standardized motor assessments: CMSA-Arm^19^, CMSA-Hand^19^, and the ARAT^20^. The CMSA Arm and Hand assesses the degree of motor impairment in the arm and hand, respectively, is defined by seven stages, ranging from 1 (flaccid paralysis) to 7 (normal movement). We summed the CMSA-Arm and CMSA-Hand stages for each participant to obtain a single representative measure of motor impairment (CMSA-Motor); we do not aim to dissociate between motor outcome for the arm and hand separately. Thus, values for CMSA-Motor range from 2 (flaccid paralysis in the arm and hand) to 14 (normal movement in the arm and hand). The ARAT is an assessment that measures the degree of motor functioning in the upper limb, and is scored from 0 (no motor function) to 57 (normal motor function)^20^.

### MRI Protocol

Magnetic resonance images were obtained on a Siemens MAGNETOM TIM Trio 3 Tesla system (Erlangen, Germany). We acquired the following scans: three-dimensional T1-weighted high resolution anatomical, T2-weighted turbo spin echo, fluid-attenuated inversion recovery (FLAIR), and resting state functional magnetic resonance imaging (rs-fMRI) scans. Parameters for each scan are listed here. T1-weighted: TR = 2000 ms; TE = 2.63 ms; flip angle = 9°; voxel size = 1 × 1 × 1 mm^3^; field of view (FOV) = 192 × 256 mm^2^; matrix size = 192 × 256 voxels; 160 axial slices; slice thickness = 1 mm. T2-weighted: TR = 2900 ms; TE 1 = 19 ms; TE 2 = 102 ms; flip angle = 180°; voxel size = 0.9 × 0.9 × 3 mm^3^; FOV = 185.68 × 220 mm^2^; matrix size = 216 × 256 voxels; 48 axial slices; slice thickness = 3 mm. FLAIR: TR = 9000 ms; TE = 96 ms; flip angle = 165°; voxel size = 0.9 × 0.9 × 5 mm^3^; FOV = 185.5 × 224 mm^2^; matrix size = 212 × 256 voxels; 32 axial slices; slice thickness = 5 mm. Rs-fMRI: TR = 2000 ms; TE = 27 ms; flip angle = 70°; voxel size = 3 × 3 × 3 mm^3^; 180 volumes; FOV = 192 × 192 mm^2^; matrix size = 64 × 64 voxels; 40 axial slices; slice thickness = 3 mm; gap = 0.5 mm. Participants fixated on a cross during the rs-fMRI scan. We recorded physiologic information during the rs-fMRI scan using a pulse oximeter on the finger to measure heart beat and a respiration belt tied around the participant’s chest to measure respiration.

### Data analyses

#### Stroke lesion

The imaging software ITK-SNAP^46^ was used to trace the lesions. A neurologist (KH) blinded to the study objectives and participant demographics manually traced the stroke lesions on the T1-weighted images. To verify the lesion tracings, the T2-weighted and FLAIR images for each participant were also reviewed. A lesion mask was created from the lesion tracings.

#### CST injury

CST Injury was defined by the amount of overlap between a normative CST template and the participant’s stroke lesion. The CST template was derived from the Johns Hopkins University (JHU) white-matter tractography atlas^47^, available in the FMRIB Software Library (FSL)^48^. Specifically, we created a binary mask of the right CST, with no threshold applied, from the JHU white-matter tractography atlas.

The lesion mask for each participant was registered into standard Montreal Neurological Institute (MNI) space with an affine transformation and FMRIB’s Non-Linear Image Registration Tool (FNIRT). The lesion mask was then flipped along the mid-sagittal plane, if required, such that all lesions were displayed on the right side of the MNI brain template.

Data were analyzed using FSL^48^ version 5.0. Based on the method by Pineiro *et al*.^6^, we found the transverse slice of the CST with the greatest overlap with the lesion [Fig. 2A] and performed the following calculation:

$$CST\,Injury=(\tfrac{Number\,of\,overlapping\,voxels\,between\,the\,CST\,and\,lesion\,for\,the\,transverse\,slice}{Total\,number\,of\,CST\,voxels\,for\,the\,transverse\,slice})\times 100 \% $$

Thus, values for CST Injury range from 0% (no overlap between the CST template and lesion mask with any transverse slice of the CST template) to 100% (complete overlap between the CST template and lesion mask for at least one transverse slice of the CST template). This approach takes into account the lesion location. A participant whose lesion overlaps a transverse slice of the CST with fewer voxels relative to other CST slices may exhibit a higher CST Injury since the transverse slice would have a greater proportion of CST voxels that are affected by the lesion. To yield data approximating a standard normal distribution, we transformed each participant’s CST Injury value into a standard z-score. These z-scores were inputted in the hierarchical regression analyses described below in the *Statistical Analyses*.

#### LM1-RM1 rs-connectivity

Preprocessing and Nuisance Regressors. Physiologic information (i.e., heartbeat and respiration) collected during the rs-fMRI scan was regressed from the rs-fMRI images using the RETROICOR algorithm^49^ in AFNI. Subsequent preprocessing and data analyses were performed using FSL^48^ version 5.0. Preprocessing of the rs-fMRI images was performed using the FMRI Expert Analysis Tool (FEAT). Preprocessing steps included head motion correction, slice time correction to the middle slice, spatial smoothing with a Gaussian kernel of 6 mm full width at half maximum, and high pass temporal filtering at 0.01 Hz. The Brain Extraction Tool (BET) in FSL was also used to remove non-brain tissue (e.g., skull).

Time series for nuisance regressors were extracted from the rs-fMRI data. Nuisance regressors included the time series for cerebrospinal fluid (CSF), white matter (WM), and six head motion parameters. To obtain the time series of CSF and WM, we used FMRIB’s Automated Segmentation Tool (FAST) to segment the T1-weighted image into grey matter, WM, and CSF. This enabled us to create masks of the CSF and WM, which were eroded twice to ensure that the only voxels included in these masks were voxels representing CSF and WM. After, we used FMRIB’s Linear Image Registration Tool (FLIRT) to transform the CSF and WM masks from structural to functional space. From the preprocessed rs-fMRI data we extracted the mean time series of CSF and WM from voxels within the CSF and WM masks, respectively. To obtain the time series of the six head motion parameters, we used FSL’s MCFLIRT. In another FEAT analysis, the mean time series for CSF, WM, and six head motion parameters were inputted in the general linear model (GLM) as regressors of no interest. The residual of this analysis was used to determine LM1-RM1 rs-connectivity described below in the subsection *LM1-RM1 Resting State Connectivity*.

Seed Masks. To calculate LM1-RM1 rs-connectivity, we created seed masks for LM1 and RM1. The first author of this study (TKL) created the seed masks independently from the neurologist (KH) who traced the stroke lesions. Given our interest in the motor outcome of both the arm and hand, the seed masks were derived using two peak coordinates from arm/elbow and hand/finger fMRI paradigms reported in a meta-analysis^50^ [Fig. 2B]. For the LM1 seed, the average peak coordinate for arm/elbow representation was (−28,−24, 62) [Supplementary Table S2A, coordinates in MNI space] and the average peak coordinate for hand/finger representation was (−36,−20, 56) [Supplementary Table S2B, coordinates in MNI space]. We then applied a 6 mm radius around each coordinate and added the two seed masks together to create a single LM1 seed. To create the RM1 seed mask, we flipped the peak coordinates used for the LM1 seed along the mid-sagittal plane such that the peak coordinates were on the right side of the MNI brain template [arm/elbow = (28, −24, 62); hand/finger = (36,−20, 56)]. The LM1 and RM1 seed masks were then transformed from standard MNI space to functional space using FNIRT.

LM1-RM1 Resting State Connectivity. Mean time series of LM1 and RM1 seeds were extracted from the residual rs-fMRI data, and used to compute the Pearson’s correlation (*r*-value) [Fig. 2B]. Two participants (s01 and s08) had partial overlap between their lesion and RM1 seed. For s01, 34% of the RM1 seed mask overlapped their lesion, whereas for s08, 10% of the RM1 seed mask overlapped their lesion. We included these two participants in the statistical analyses since the perilesional tissue in the right M1 region appears mostly intact for these participants. To yield data approximating a standard normal distribution, we transformed each participant’s LM1-RM1 rs-connectivity *r*-value into a standard z-score. These z-scores were inputted in the hierarchical regression analyses described in *Statistical Analyses*.

#### Statistical analyses

Hierarchical regression analyses to explain variability in motor assessment scores. We performed a series of hierarchical multiple regression analyses to model motor outcome using SPSS version 22.0. We first fit an interaction model with three terms (z-scores for CST Injury, z-scores for LM1-RM1 rs-connectivity, and their interaction) to explain the variability in the CMSA-Motor score. The interaction term was derived from multiplying the z-scores for CST Injury and LM1-RM1 rs-connectivity together for each participant. We then fit an additive model with two terms (z-scores for both CST Injury and LM1-RM1 rs-connectivity) to explain the variability in the CMSA-Motor score. Lastly, we implemented two simple regression models to explain the variability in the CMSA-Motor score; simple regression model 1 includes z-scores for CST Injury, and simple regression model 2 includes z-scores for LM1-RM1 rs-connectivity. The same procedure as above was repeated to fit an interaction, additive, and two simple regression models to explain the variability in the ARAT score. We verified that the residuals of each model were approximately normally distributed using the Shapiro-Wilk statistic (*p* < 0.05 indicates that the residuals are not normally distributed) and through visual inspection of the histogram of the residuals. We report the R^2^, adjusted R^2^, F-statistic, and beta (β)-values for each term in all four models (i.e., interaction, additive, and two simple regressions).

To address our main objective, we calculated the ΔR^2^ value to determine whether the interaction model is significantly different from the additive model. The ΔR^2^ value represents the difference in the amount of explained variability between the two models. If the ΔR^2^ value is significant (*p* < 0.05), the interaction term significantly contributes to explaining variability in motor outcome. We calculated a Pearson’s correlation between CST Injury and LM1-RM1 rs-connectivity to determine the relationship between these biomarkers. If the interaction term is not significant, we then calculated the ΔR^2^ value to determine whether the additive model significantly differs from either simple regression models.

Supplementary analyses. To verify that our results are not influenced by factors associated with participant demographics, we also performed hierarchical regression analyses that included the following covariates: age, sex, time since stroke (months), years of education, and dominant hand affected. We did not include lesion volume as a covariate in the hierarchical regression analyses since CST Injury and lesion volume are likely collinear. We selected CST Injury, rather than lesion volume, as a biomarker of the structural integrity of the motor system since the former is a better predictor of motor recovery after stroke than the latter^7,8,51,52^. We assessed the influence of each covariate separately given statistical power limitations with a small sample. We assessed the R^2^, adjusted R^2^, and β-values for each model. We also calculated the ΔR^2^ to assess whether the interaction model is significantly different from the additive model.

To control for participants with bilateral (N = 3) or cerebellar (N = 2) lesions who may show different clinical presentations from those with unilateral and cortical/subcortical lesions, we performed additional analyses excluding these participants in the hierarchical regression models.

### Data availability

The data generated and analyzed during this study are included in this published article (see Supplementary Table S1).

## Electronic supplementary material

Supplementary Materials
