# Supplementary material for: Variability in stroke motor outcome is explained by structural and functional integrity of the motor system
Source: Sci Rep. 2018 Jun 21;8:9480. doi: 10.1038/s41598-018-27541-8 (PMC6013462; doi:10.1038/s41598-018-27541-8)
Supplement: Supplementary file 1 — Supplementary Materials [file 41598_2018_27541_MOESM1_ESM.docx]

**Supplementary Materials**

**Variability in stroke motor outcome is explained by structural and functional integrity of the motor system**

Timothy K. Lam^1,2,3^, Malcolm A. Binns^4,5^, Kie Honjo^1,2^, Deirdre R. Dawson^1,3,4,6^, Bernhard Ross^4,7^, Donald T. Stuss^4,8,9^, Sandra E. Black^1,2,3,4,9^, J. Jean Chen^4,7^, Takako Fujioka^4,10^, Joyce L. Chen*^1,2,3,11^

^1^Heart and Stroke Foundation Canadian Partnership for Stroke Recovery, Toronto, ON, Canada

^2^Hurvitz Brain Sciences Research Program, Sunnybrook Research Institute, Toronto, ON, Canada

^3^Rehabilitation Sciences Institute, University of Toronto, Toronto, ON, Canada

^4^Rotman Research Institute, Baycrest Centre, Toronto, ON, Canada

^5^Dalla Lana School of Public Health, University of Toronto, Toronto, ON, Canada

^6^Department of Occupational Science and Occupational Therapy, University of Toronto, Toronto, ON, Canada

^7^Department of Medical Biophysics, University of Toronto, Toronto, ON, Canada

^8^Department of Psychology, University of Toronto, Toronto, ON, Canada

^9^Department of Medicine (Neurology), University of Toronto, Toronto, ON, Canada

^10^Center for Computer Research in Music and Acoustics, Department of Music, Stanford Neurosciences Institute, Stanford University, Stanford, CA, USA

^11^Department of Physical Therapy, University of Toronto, Toronto, ON, Canada

***Corresponding Author**:

Joyce L. Chen, PhD, Sunnybrook Research Institute, 2075 Bayview Avenue, Room M6-176, Toronto, ON, M4N 3M5, Canada, (T) 416-480-6100 x85410, (F) 416-480-4223, E-mail: [joyce.chen@sunnybrook.ca](mailto:joyce.chen@sunnybrook.ca)

**Supplementary Table S1: Individual data (participant demographics, performance on motor assessments, and neurological measures)**

| **Participant** | **Age (years)** | **Sex (M/F)** | **Time since stroke (months)** | **Education (years)** | **CMSA-Arm**  **(score)** | **CMSA-Hand (score)** | **CMSA-Motor (score)** | **ARAT (score)** | **CST Injury**  **(percent)** | **LM1-RM1 rs-connectivity**  **(*r*-value)** |
| --- | --- | --- | --- | --- | --- | --- | --- | --- | --- | --- |
| 1 | 76.42 | F | 86 | 14 | 2 | 2 | 4 | 9 | 100 | 0.32 |
| 2 | 64.25 | F | 14 | 16 | 4 | 4 | 8 | 49 | 36.36 | 0.80 |
| 3 | 73.92 | M | 20 | 12 | 4 | 4 | 8 | 50 | 9.33 | 0.17 |
| 4 | 79.17 | M | 20 | 10 | 5 | 5 | 10 | 48 | 3.03 | 0.62 |
| 5 | 44.83 | M | 34 | 17 | 3 | 3 | 6 | 37 | 13.79 | 0.44 |
| 6 | 72.42 | M | 63 | 19 | 3 | 4 | 7 | 26 | 19.64 | 0.44 |
| 7 | 61.83 | M | 218 | 17 | 2 | 2 | 4 | 6 | 30.67 | 0.21 |
| 8 | 55.67 | F | 71 | 14 | 3 | 3 | 6 | 13 | 100 | 0.48 |
| 9 | 54.08 | M | 13 | 22 | 3 | 3 | 6 | 6 | 65.33 | 0.65 |
| 10 | 55.92 | M | 34 | 12 | 4 | 4 | 8 | 57 | 1.67 | 0.30 |
| 11 | 47.17 | M | 9 | 16 | 4 | 2 | 6 | 31 | 78.85 | 0.45 |
| 12 | 67.83 | M | 7 | 18 | 3 | 4 | 7 | 39 | 46.67 | 0.62 |
| 13 | 73.17 | M | 22 | 16 | 2 | 2 | 4 | 0 | 58.18 | 0.30 |
| 14 | 53.58 | M | 33 | 15 | 2 | 2 | 4 | 0 | 47.46 | 0.38 |
| 15 | 56.92 | M | 46 | 16 | 3 | 5 | 8 | 53 | 47.06 | 0.79 |
| 16 | 41.58 | F | 305 | 18 | 3 | 2 | 5 | 4 | 84.75 | 0.36 |
| 17 | 66.50 | M | 59 | 15 | 4 | 4 | 8 | 48 | 26.67 | 0.33 |
| 18 | 71.17 | F | 99 | 11 | 3 | 4 | 7 | 41 | 74.24 | 0.33 |
| 19 | 63.67 | M | 11 | 13 | 3 | 2 | 5 | 4 | 22.22 | 0.18 |
| 20 | 54.92 | F | 50 | 20 | 3 | 4 | 7 | 43 | 37.29 | 0.41 |
| 21 | 67.42 | M | 138 | 16 | 3 | 2 | 5 | 0 | 48.57 | 0.42 |
| 22 | 53.83 | F | 12 | 17 | 2 | 2 | 4 | 2 | 48.48 | 0.23 |
| 23 | 75.33 | F | 64 | 12 | 4 | 4 | 8 | 55 | 28.87 | 0.60 |
| 24 | 58.42 | M | 47 | 19 | 4 | 5 | 9 | 49 | 58.45 | 0.72 |
| 25 | 59.58 | M | 22 | 16 | 3 | 2 | 5 | 11 | 69.12 | 0.19 |

**Abbreviations**: Sex (Male/Female); CMSA-Arm (Chedoke-McMaster Stroke Assessment Impairment Inventory: Stage of Arm Impairment, 1-7); CMSA-Hand (Chedoke-McMaster Stroke Assessment Impairment Inventory: Stage of Hand Impairment, 1-7); CMSA-Motor (composite measure of the CMSA-Arm and CMSA-Hand stages, 2-14); ARAT (Action Research Arm Test, 0-57); CST Injury (percent injury to the corticospinal tract); LM1-RM1 rs-connectivity (resting state connectivity between left and right primary motor cortex).

**Supplementary Table S2: Peak coordinates averaged for left primary motor cortex (M1) seed based on studies with an arm/elbow or hand/finger paradigm**

*Supplementary Table S2A: Arm/Elbow paradigm*

| First Author (Year) *Journal* | Paradigm | x | y | z |
| --- | --- | --- | --- | --- |
| Alkadhi (2002) *Am J Neuroradiol* | Elbow Movement | -29 | -25 | 61 |
| Lotze (2000) *Neuroreport* | Elbow Movement | -28 | -24 | 64 |
|  |  |  |  |  |
| **Average Coordinates** |  | -28 | -24 | 62 |

All peak coordinates are from healthy participants.

*Supplementary Table S2B: Hand/Finger paradigm*

| First Author (Year) *Journal* | Paradigm | x | y | z |
| --- | --- | --- | --- | --- |
| Adam (2003) *Cogn Brain Res* | Finger Key Press | -34 | -14 | 62 |
| Alkadhi (2002) *Am J Neuroradiol* | Hand Movement &  Finger Movement | -36.5 | -21 | 58 |
| Baraldi (1999) *Neurosci Lett* | Finger Movement | -31 | -15 | 51 |
| Bischoff-Grethe (2002) *J Neurosci* | Button Press | -30 | -28 | 42 |
| Calhoun (2002) *Hum Brain Mapp* | Hand Driving | -33 | -13 | 60 |
| Choi (2001) *Exp Brain Res* | Hand Gestures |  |  |  |
| Culham (2003) *Exp Brain Res* | Grasp vs. Reach | -29 | -18 | 56 |
| Cunnington (2002) *Neuroimage* | Finger Press | -41 | -17 | 60 |
| De Weerd (2003) *Neuroimage* | Hand Tapping | -42 | -17 | 49 |
| Debaere (2001) *Neuroimage* | Wrist Flexion/Extension | -40.5 | -24 | 64.5 |
| Dhamala (2003) *Neuroimage* | Rhythmic Finger Tapping | -36 | -28 | 64 |
| Dreher (2002) *Eur J Neurosci* | Button Press | -28 | -16 | 60 |
| Ehrsson (2000) *J Neurophysiol* | Precision &  Power Grip | -34 | -20 | 56 |
| Ehrsson (2001) *J Neurophysiol* | Grip | -36 | -20 | 48 |
| Ehrsson (2003) *J Neurophysiol* | Finger Grip | -40 | -36 | 60 |
| Foltys (2003) *Neuroimage* | Hand Clenching | -33 | -29 | 56 |
| Hamzei (2002) *Exp Brain Res* | Writing | -24 | -16 | 54 |
| Hamzei (2002) *Neuroimage* | Pinch Grip | -42 | -12 | 58 |
| Haslinger (2002) *Cogn Brain Res* | Finger Movement | -42 | -19 | 56 |
| Indovina (2001) *Exp Brain Res* | Button Press | -38 | -20 | 68 |
| Indovina (2001) *Neuroimage* | Finger Movement | -34.4 | -26.4 | 57.5 |
| Ino (2003) *Neurosci Res* | Clock Drawing | -34 | -12 | 56 |
| Jancke (2000) *Cogn Brain Res* | Finger Tapping | -49 | -20 | 49 |
| Jantzen (2002) *Neurosci Lett* | Finger Flexion | -33.5 | -17 | 57 |
| Johansen-Berg (2002) *Exp Brain Res* | Button Pressing | -28 | -8 | 54 |
| Kawashima (2000) *J Neurophsyiol* | Visually Cued Finger Movement | -34 | -18 | 60 |
| Kobayashi (2003) *Neuroimage* | Finger Movement | -40.8 | -17.2 | 57.4 |
| Koski (2002) *Cereb Cortex* | Finger Movement | -40 | -24 | 58 |
| Kuhtz-Buschbeck (2001) *Eur J Neurosci* | Natural Grip | -40 | -24 | 52 |
| Liddle (2001) *Hum Brain Mapp* | Finger Press | -38 | -26 | 65 |
| Lotze (2000) *Neuroreport* | Finger Tap | -40 | -16 | 56 |
| Lotze (2003) *Brain* | Voluntary Wrist Movement | -38 | -20 | 58 |
| Matsuo (2003) *Cogn Brain Res* | Finger Movement | -38 | -18 | 58 |
| Mattay (1998) *Psychiatry Res: Neuroimaging Sect* | Thumb Opposition | -36 | -22 | 68 |
| Mayville (2002) *Hum Brain Mapp* | Pinch | -39 | -28 | 56 |
| Muller (2002) *Cogn Brain Res* | Finger Tapping | -33 | -21 | 60 |
| Rao (1997) *J Neurosci* | Finger Tapping | -35 | -24 | 55 |
| Riecker (2003) *Neuroimage* | Finger Tapping | -45 | -24 | 60 |
| Rotte (2002) *Stereotact Funct Neurosurg* | Finger Movement | -35 | -18 | 59 |
| Rowe (2002) *Brain* | Finger Tapping | -50 | -28 | 48 |
| Sakai (2000) *J Neurosci* | Button Press | -36 | -16 | 46 |
| Schubotz (2001) *Cereb Cortex* | Finger Tapping | -35 | -19 | 61 |
| Stippich (2002) *Neurosci Lett* | Hand Movement | -37.14 | -24.5 | 55.21 |
| Stoeckel (2003) *Neuroimage* | Hand Movement | -44 | -17 | 43 |
| Sugio (2003) *Neuroreport* | Grasp | -32 | -20.5 | 53.5 |
| Toma (2003) *Neurosci Lett* | Button Press | -44 | -15 | 50 |
| Toni (1998) *Neuroimage* | Finger Movements | -40 | -18 | 64 |
| Toni (2002) *J Cogn Neurosci* | Finger Press | -26 | -22 | 66 |
| Vaillancourt (2003) *J Neurophysiol* | Grip | -15 | -16 | 63 |
| Watanabe (2002) *Neuroimage* | Finger Press | -32 | -8 | 62 |
| Winterer (2002) *Neuroimage* | Button Press | -35 | -26 | 50 |
|  |  |  |  |  |
| **Average Coordinates** |  | -36 | -20 | 56 |

All peak coordinates are from healthy participants.

**Supplementary Table S3: Hierarchical Multiple Regression for CMSA-Motor and ARAT, including age as a covariate**

|  | **R^2^** | **Adjusted R^2^** | ***p*-value** | **β** | ***p*-value** | **ΔR^2^** | ***p*-value** |
| --- | --- | --- | --- | --- | --- | --- | --- |
| ***Chedoke-McMaster Stroke Assessment Impairment Inventory Total Motor Impairment (CMSA-Motor)*** | | | | | | | |
| **Interaction Model**  CST Injury  LM1-RM1 rs-connectivity  Interaction  Age | 0.59 | 0.51 | 0.001 | –0.42  0.63  0.12  0.21 | 0.01  0.001  0.47  0.19 | - | - |
| **Additive Model**  CST Injury  LM1-RM1 rs-connectivity  Age | 0.58 | 0.52 | < 0.001 | –0.44  0.58  0.17 | 0.007  < 0.001  0.25 | –0.01^#^ | 0.47 |
| **Simple Regression 1**  CST Injury  Age | 0.24 | 0.17 | 0.05 | –0.42  0.17 | 0.03  0.39 | –0.34^ | < 0.001 |
| **Simple Regression 2**  LM1-RM1 rs-connectivity  Age | 0.40 | 0.34 | 0.004 | 0.57  0.28 | 0.002  0.11 | –0.18^ | 0.007 |
|  |  |  |  |  |  |  |  |
| ***Action Research Arm Test (ARAT)*** | | | | | | | |
| **Interaction Model**  CST Injury  LM1-RM1 rs-connectivity  Interaction  Age | 0.45 | 0.34 | 0.01 | –0.45  0.50  0.08  0.12 | 0.01  0.01  0.67  0.51 | - | - |
| **Additive Model**  CST Injury  LM1-RM1 rs-connectivity  Age | 0.45 | 0.37 | 0.005 | –0.46  0.47  0.09 | 0.01  0.009  0.58 | –0.005^#^ | 0.67 |
| **Simple Regression 1**  CST Injury  Age | 0.23 | 0.16 | 0.05 | –0.45  0.09 | 0.02  0.64 | –0.22^ | 0.009 |
| **Simple Regression 2**  LM1-RM1 rs-connectivity  Age | 0.25 | 0.18 | 0.04 | 0.45  0.21 | 0.02  0.28 | –0.20^ | 0.01 |

R^2^, adjusted R^2^, β-values, ΔR^2^ values, and the associated significance (*p*-values) for the hierarchical multiple regression models to explain variability in performance on motor assessments, with age included as a covariate in the models. Hash (^#^) represents the ΔR^2^ value from the comparison between the interaction model and additive model. Caret (^) represents the ΔR^2^ value from the comparison between the additive model and simple regression model. Model comparisons are considered significant at *p*<0.05.

**Supplementary Table S4: Hierarchical Multiple Regression for CMSA-Motor and ARAT, including sex as a covariate**

|  | **R^2^** | **Adjusted R^2^** | ***p*-value** | **β** | ***p*-value** | **ΔR^2^** | ***p*-value** |
| --- | --- | --- | --- | --- | --- | --- | --- |
| ***Chedoke-McMaster Stroke Assessment Impairment Inventory Total Motor Impairment (CMSA-Motor)*** | | | | | | | |
| **Interaction Model**  CST Injury  LM1-RM1 rs-connectivity  Interaction  Sex | 0.57 | 0.48 | 0.001 | –0.53  0.62  0.09  0.14 | 0.004  0.001  0.59  0.41 | - | - |
| **Additive Model**  CST Injury  LM1-RM1 rs-connectivity  Sex | 0.56 | 0.50 | < 0.001 | –0.53  0.58  0.12 | 0.003  0.001  0.48 | –0.006^#^ | 0.59 |
| **Simple Regression 1**  CST Injury  Sex | 0.22 | 0.16 | 0.05 | –0.52  0.13 | 0.02  0.53 | –0.34^ | 0.001 |
| **Simple Regression 2**  LM1-RM1 rs-connectivity  Sex | 0.33 | 0.28 | 0.01 | 0.57  –0.12 | 0.003  0.51 | –0.23^ | 0.003 |
|  |  |  |  |  |  |  |  |
| ***Action Research Arm Test (ARAT)*** | | | | | | | |
| **Interaction Model**  CST Injury  LM1-RM1 rs-connectivity  Interaction  Sex | 0.49 | 0.39 | 0.007 | –0.59  0.51  0.12  0.27 | 0.003  0.009  0.52  0.17 | - | - |
| **Additive Model**  CST Injury  LM1-RM1 rs-connectivity  Sex | 0.48 | 0.41 | 0.003 | –0.59  0.46  0.23 | 0.003  0.008  0.20 | –0.01^#^ | 0.52 |
| **Simple Regression 1**  CST Injury  Sex | 0.27 | 0.20 | 0.03 | –0.58  0.24 | 0.009  0.24 | –0.21^ | 0.008 |
| **Simple Regression 2**  LM1-RM1 rs-connectivity  Sex | 0.20 | 0.13 | 0.08 | 0.45  –0.02 | 0.02  0.89 | –0.28^ | 0.003 |

R^2^, adjusted R^2^, β-values, ΔR^2^ values, and the associated significance (*p*-values) for the hierarchical multiple regression models to explain variability in performance on motor assessments, with sex included as a covariate in the models. Hash (^#^) represents the ΔR^2^ value from the comparison between the interaction model and additive model. Caret (^) represents the ΔR^2^ value from the comparison between the additive model and simple regression model. Model comparisons are considered significant at *p*<0.05.

**Supplementary Table S5: Hierarchical Multiple Regression for CMSA-Motor and ARAT, including time since stroke as a covariate**

|  | **R^2^** | **Adjusted R^2^** | ***p*-value** | **β** | ***p*-value** | **ΔR^2^** | ***p*-value** |
| --- | --- | --- | --- | --- | --- | --- | --- |
| ***Chedoke-McMaster Stroke Assessment Impairment Inventory Total Motor Impairment (CMSA-Motor)*** | | | | | | | |
| **Interaction Model**  CST Injury  LM1-RM1 rs-connectivity  Interaction  Time since stroke | 0.56 | 0.47 | 0.002 | –0.46  0.59  0.05  –0.04 | 0.008  0.002  0.78  0.80 | - | - |
| **Additive Model**  CST Injury  LM1-RM1 rs-connectivity  Time since stroke | 0.56 | 0.49 | 0.001 | –0.47  0.57  –0.04 | 0.006  0.001  0.78 | –0.002^#^ | 0.78 |
| **Simple Regression 1**  CST Injury  Time since stroke | 0.25 | 0.17 | 0.04 | –0.41  –0.17 | 0.04  0.38 | –0.31^ | 0.001 |
| **Simple Regression 2**  LM1-RM1 rs-connectivity  Time since stroke | 0.36 | 0.29 | 0.008 | 0.53  –0.18 | 0.006  0.31 | –0.20^ | 0.006 |
|  |  |  |  |  |  |  |  |
| ***Action Research Arm Test (ARAT)*** | | | | | | | |
| **Interaction Model**  CST Injury  LM1-RM1 rs-connectivity  Interaction  Time since stroke | 0.45 | 0.34 | 0.01 | –0.46  0.46  0.03  –0.08 | 0.01  0.02  0.85  0.64 | - | - |
| **Additive Model**  CST Injury  LM1-RM1 rs-connectivity  Time since stroke | 0.45 | 0.37 | 0.005 | –0.46  0.45  –0.08 | 0.01  0.01  0.62 | –0.004^#^ | 0.69 |
| **Simple Regression 1**  CST Injury  Time since stroke | 0.26 | 0.19 | 0.03 | –0.42  –0.19 | 0.03  0.33 | –0.19^ | 0.01 |
| **Simple Regression 2**  LM1-RM1 rs-connectivity  Time since stroke | 0.25 | 0.18 | 0.04 | 0.41  –0.22 | 0.04  0.25 | –0.20^ | 0.01 |

R^2^, adjusted R^2^, β-values, ΔR^2^ values, and the associated significance (*p*-values) for the hierarchical multiple regression models to explain variability in performance on motor assessments, with time since stroke (in months) included as a covariate in the models. Hash (^#^) represents the ΔR^2^ value from the comparison between the interaction model and additive model. Caret (^) represents the ΔR^2^ value from the comparison between the additive model and simple regression model. Model comparisons are considered significant at *p*<0.05.

**Supplementary Table S6: Hierarchical Multiple Regression for CMSA-Motor and ARAT, including years of education as a covariate**

|  | **R^2^** | **Adjusted R^2^** | ***p*-value** | **β** | ***p*-value** | **ΔR^2^** | ***p*-value** |
| --- | --- | --- | --- | --- | --- | --- | --- |
| ***Chedoke-McMaster Stroke Assessment Impairment Inventory Total Motor Impairment (CMSA-Motor)*** | | | | | | | |
| **Interaction Model**  CST Injury  LM1-RM1 rs-connectivity  Interaction  Years of education | 0.66 | 0.59 | < 0.001 | –0.38  0.73  0.15  –0.35 | 0.01  < 0.001  0.32  0.02 | - | - |
| **Additive Model**  CST Injury  LM1-RM1 rs-connectivity  Years of education | 0.64 | 0.59 | < 0.001 | –0.41  0.66  –0.31 | 0.006  < 0.001  0.03 | –0.02^#^ | 0.32 |
| **Simple Regression 1**  CST Injury  Years of education | 0.23 | 0.17 | 0.05 | –0.43  –0.15 | 0.03  0.44 | –0.41^ | < 0.001 |
| **Simple Regression 2**  LM1-RM1 rs-connectivity  Years of education | 0.48 | 0.44 | 0.001 | 0.67  –0.41 | < 0.001  0.02 | –0.16^ | 0.006 |
|  |  |  |  |  |  |  |  |
| ***Action Research Arm Test (ARAT)*** | | | | | | | |
| **Interaction Model**  CST Injury  LM1-RM1 rs-connectivity  Interaction  Years of education | 0.55 | 0.46 | 0.002 | –0.39  0.61  0.15  –0.36 | 0.02  0.002  0.40  0.04 | - | - |
| **Additive Model**  CST Injury  LM1-RM1 rs-connectivity  Years of education | 0.53 | 0.47 | 0.001 | –0.41  0.54  –0.32 | 0.01  0.002  0.05 | –0.02^#^ | 0.40 |
| **Simple Regression 1**  CST Injury  Years of education | 0.25 | 0.19 | 0.03 | –0.43  –0.19 | 0.03  0.32 | –0.28^ | 0.002 |
| **Simple Regression 2**  LM1-RM1 rs-connectivity  Years of education | 0.37 | 0.32 | 0.006 | 0.55  –0.42 | 0.004  0.02 | –0.16^ | 0.01 |

R^2^, adjusted R^2^, β-values, ΔR^2^ values, and the associated significance (*p*-values) for the hierarchical multiple regression models to explain variability in performance on motor assessments, with years of education included as a covariate in the models. Hash (^#^) represents the ΔR^2^ value from the comparison between the interaction model and additive model. Caret (^) represents the ΔR^2^ value from the comparison between the additive model and simple regression model. Model comparisons are considered significant at *p*<0.05.

**Supplementary Table S7: Hierarchical Multiple Regression for CMSA-Motor and ARAT, including dominant hand affected as a covariate**

|  | **R^2^** | **Adjusted R^2^** | ***p*-value** | **β** | ***p*-value** | **ΔR^2^** | ***p*-value** |
| --- | --- | --- | --- | --- | --- | --- | --- |
| ***Chedoke-McMaster Stroke Assessment Impairment Inventory Total Motor Impairment (CMSA-Motor)*** | | | | | | | |
| **Interaction Model**  CST Injury  LM1-RM1 rs-connectivity  Interaction  Dominant hand affected | 0.53 | 0.44 | 0.004 | –0.49  0.62  0.03  –0.13 | 0.008  0.002  0.86  0.41 | - | - |
| **Additive Model**  CST Injury  LM1-RM1 rs-connectivity  Dominant hand affected | 0.53 | 0.46 | 0.001 | –0.49  0.61  –0.14 | 0.006  0.001  0.38 | –0.001^#^ | 0.86 |
| **Simple Regression 1**  CST Injury  Dominant hand affected | 0.17 | 0.09 | 0.14 | –0.43  –0.12 | 0.05  0.58 | –0.36^ | 0.001 |
| **Simple Regression 2**  LM1-RM1 rs-connectivity  Dominant hand affected | 0.31 | 0.25 | 0.01 | 0.56  –0.01 | 0.005  0.94 | –0.22^ | 0.006 |
|  |  |  |  |  |  |  |  |
| ***Action Research Arm Test (ARAT)*** | | | | | | | |
| **Interaction Model**  CST Injury  LM1-RM1 rs-connectivity  Interaction  Dominant hand affected | 0.43 | 0.31 | 0.02 | –0.50  0.51  0.07  –0.03 | 0.01  0.01  0.73  0.86 | - | - |
| **Additive Model**  CST Injury  LM1-RM1 rs-connectivity  Dominant hand affected | 0.43 | 0.34 | 0.009 | –0.50  0.48  –0.04 | 0.01  0.01  0.82 | –0.004^#^ | 0.73 |
| **Simple Regression 1**  CST Injury  Dominant hand affected | 0.20 | 0.12 | 0.09 | –0.45  –0.02 | 0.03  0.92 | –0.23^ | 0.01 |
| **Simple Regression 2**  LM1-RM1 rs-connectivity  Dominant hand affected | 0.20 | 0.12 | 0.09 | 0.44  0.09 | 0.03  0.66 | –0.23^ | 0.01 |

R^2^, adjusted R^2^, β-values, ΔR^2^ values, and the associated significance (*p*-values) for the hierarchical multiple regression models to explain variability in performance on motor assessments, with whether the participant’s dominant hand affected included as a covariate in the models. Hash (^#^) represents the ΔR^2^ value from the comparison between the interaction model and additive model. Caret (^) represents the ΔR^2^ value from the comparison between the additive model and simple regression model. Model comparisons are considered significant at *p*<0.05.

**Supplementary Table S8:** **Hierarchical Multiple Regression for CMSA-Motor and ARAT, after removing participants with bilateral lesions (N = 22)**

|  | **R^2^** | **Adjusted R^2^** | ***p*-value** | **β** | ***p*-value** | **ΔR^2^** | ***p*-value** |
| --- | --- | --- | --- | --- | --- | --- | --- |
| ***Chedoke-McMaster Stroke Assessment Impairment Inventory Total Motor Impairment (CMSA-Motor)*** | | | | | | | |
| **Interaction Model**  CST Injury  LM1-RM1 rs-connectivity  Interaction | 0.58 | 0.51 | 0.001 | –0.52  0.59  0.04 | 0.003  0.002  0.82 | - | - |
| **Additive Model**  CST Injury  LM1-RM1 rs-connectivity | 0.58 | 0.54 | < 0.001 | –0.52  0.58 | 0.002  0.001 | –0.001^#^ | 0.82 |
| **Simple Regression 1**  CST Injury | 0.25 | 0.21 | 0.02 | –0.50 | 0.02 | –0.33^ | 0.001 |
| **Simple Regression 2**  LM1-RM1 rs-connectivity | 0.31 | 0.27 | 0.007 | 0.56 | 0.007 | –0.27^ | 0.002 |
|  |  |  |  |  |  |  |  |
| ***Action Research Arm Test (ARAT)*** | | | | | | | |
| **Interaction Model**  CST Injury  LM1-RM1 rs-connectivity  Interaction | 0.43 | 0.34 | 0.01 | –0.51  0.44  –0.03 | 0.01  0.03  0.87 | - | - |
| **Additive Model**  CST Injury  LM1-RM1 rs-connectivity | 0.43 | 0.37 | 0.005 | –0.51  0.43 | 0.008  0.02 | –0.001^#^ | 0.87 |
| **Simple Regression 1**  CST Injury | 0.24 | 0.21 | 0.05 | –0.49 | 0.02 | –0.19^ | 0.02 |
| **Simple Regression 2**  LM1-RM1 rs-connectivity | 0.17 | 0.13 | 0.05 | 0.41 | 0.05 | –0.26^ | 0.008 |

R^2^, adjusted R^2^, β-values, ΔR^2^ values, and the associated significance (*p*-values) for the hierarchical multiple regression models to explain variability in performance on motor assessments after removing participants with bilateral lesions. Hash (^#^) represents the ΔR^2^ value from the comparison between the interaction model and additive model. Caret (^) represents the ΔR^2^ value from the comparison between the additive model and simple regression model. Model comparisons are considered significant at *p*<0.05.

**Supplementary Table S9: Hierarchical Multiple Regression for CMSA-Motor and ARAT, after removing participants with cerebellar lesions (N = 23)**

|  | **R^2^** | **Adjusted R^2^** | ***p*-value** | **β** | ***p*-value** | **ΔR^2^** | ***p*-value** |
| --- | --- | --- | --- | --- | --- | --- | --- |
| ***Chedoke-McMaster Stroke Assessment Impairment Inventory Total Motor Impairment (CMSA-Motor)*** | | | | | | | |
| **Interaction Model**  CST Injury  LM1-RM1 rs-connectivity  Interaction | 0.52 | 0.44 | 0.003 | –0.52  0.51  –0.004 | 0.004  0.01  0.98 | - | - |
| **Additive Model**  CST Injury  LM1-RM1 rs-connectivity | 0.52 | 0.47 | 0.001 | –0.52  0.51 | 0.003  0.004 | 0.00^#^ | 0.98 |
| **Simple Regression 1**  CST Injury | 0.26 | 0.22 | 0.01 | –0.51 | 0.01 | –0.26^ | 0.004 |
| **Simple Regression 2**  LM1-RM1 rs-connectivity | 0.25 | 0.21 | 0.02 | 0.49 | 0.02 | –0.27^ | 0.003 |
|  |  |  |  |  |  |  |  |
| ***Action Research Arm Test (ARAT)*** | | | | | | | |
| **Interaction Model**  CST Injury  LM1-RM1 rs-connectivity  Interaction | 0.40 | 0.30 | 0.02 | –0.50  0.40  0.03 | 0.01  0.05  0.88 | - | - |
| **Additive Model**  CST Injury  LM1-RM1 rs-connectivity | 0.40 | 0.34 | 0.006 | –0.50  0.39 | 0.009  0.03 | –0.001^#^ | 0.88 |
| **Simple Regression 1**  CST Injury | 0.24 | 0.21 | 0.02 | –0.49 | 0.02 | –0.16^ | 0.03 |
| **Simple Regression 2**  LM1-RM1 rs-connectivity | 0.14 | 0.10 | 0.07 | 0.38 | 0.07 | –0.26^ | 0.009 |

R^2^, adjusted R^2^, β-values, ΔR^2^ values, and the associated significance (*p*-values) for the hierarchical multiple regression models to explain variability in performance on motor assessments after removing participants with cerebellar lesions. Hash (^#^) represents the ΔR^2^ value from the comparison between the interaction model and additive model. Caret (^) represents the ΔR^2^ value from the comparison between the additive model and simple regression model. Model comparisons are considered significant at *p*<0.05.

**Supplementary Table S10: Hierarchical Multiple Regression for CMSA-Motor and ARAT, after removing participants with partial right primary motor cortex (RM1) seed overlap (N=23)**

|  | **R^2^** | **Adjusted R^2^** | ***p*-value** | **β** | ***p*-value** | **ΔR^2^** | ***p*-value** |
| --- | --- | --- | --- | --- | --- | --- | --- |
| ***Chedoke-McMaster Stroke Assessment Impairment Inventory Total Motor Impairment (CMSA-Motor)*** | | | | | | | |
| **Interaction Model**  CST Injury  LM1-RM1 rs-connectivity  Interaction | 0.53 | 0.46 | 0.002 | –0.46  0.61  0.01 | 0.009  0.001  0.93 | - | - |
| **Additive Model**  CST Injury  LM1-RM1 rs-connectivity | 0.53 | 0.48 | 0.001 | –0.46  0.60 | 0.007  0.001 | 0^#^ | 0.25 |
| **Simple Regression 1**  CST Injury | 0.17 | 0.14 | 0.04 | –0.42 | 0.04 | –0.36^ | 0.009 |
| **Simple Regression 2**  LM1-RM1 rs-connectivity | 0.32 | 0.29 | 0.005 | 0.56 | 0.005 | –0.21^ | 0.02 |
|  |  |  |  |  |  |  |  |
| ***Action Research Arm Test (ARAT)*** | | | | | | | |
| **Interaction Model**  CST Injury  LM1-RM1 rs-connectivity  Interaction | 0.42 | 0.33 | 0.01 | –0.46  0.51  0.06 | 0.02  0.02  0.76 | - | - |
| **Additive Model**  CST Injury  LM1-RM1 rs-connectivity | 0.42 | 0.36 | 0.004 | –0.46  0.49 | 0.01  0.01 | –0.003^#^ | 0.76 |
| **Simple Regression 1**  CST Injury | 0.18 | 0.14 | 0.04 | –0.52 | 0.004 | –0.24^ | 0.01 |
| **Simple Regression 2**  LM1-RM1 rs-connectivity | 0.20 | 0.17 | 0.03 | 0.45 | 0.03 | –0.22^ | 0.01 |

R^2^, adjusted R^2^, β-values, ΔR^2^ values, and the associated significance (*p*-values) for the hierarchical multiple regression models to explain variability in performance on motor assessments, after removing participants who have their lesion overlapping the M1 seed. Hash (^#^) represents the ΔR^2^ value from the comparison between the interaction model and additive model. Caret (^) represents the ΔR^2^ value from the comparison between the additive model and simple regression model. Model comparisons are considered significant at *p*<0.05.

**Supplementary References**

1. Adam, J. J. *et al*. Rapid visuomotor preparation in the human brain: a functional MRI study. *Cogn. Brain Res.* **16**, 1-10 (2003).
2. Alkadhi, H. *et al*. Reproducibility of primary motor cortex somatotopy under controlled conditions. *Am. J. Neuroradiol.* **23**, 1524-1532 (2002).
3. Baraldi, P. *et al*. Bilateral representation of sequential finger movements in human cortical areas. *Neurosci. Lett.* **269**, 95-98 (1999).
4. Bischoff-Grethe, A., Ivry, R. B. & Grafton, S. T. Cerebellar involvement in response reassignment rather than attention. *J. Neurosci.* **22**, 546-553 (2002).
5. Calhoun, V. D. *et al*. Different activation dynamics in multiple neural systems during simulated driving. *Hum. Brain Mapp.* **17**, 141-142 (2002).
6. Choi, S. H. *et al*. Functional magnetic resonance imaging during pantomiming tool-use gestures. *Exp. Brain Res.* **139**, 311-317 (2001).
7. Culham, J. C. *et al*. Visually guided grasping produces fMRI activation in dorsal but not ventral stream brain areas. *Exp. Brain Res.* **153**, 180-189 (2003).
8. Cunnington, R., Windischberger, C., Deecke, L. & Moser, E. The preparation and execution of self-initiated and externally-triggered movement: a study of event-related fMRI. *Neuroimage* **15**, 373-385 (2002).
9. De Weerd, P. *et al*. Cortical mechanisms for acquisition and performance of bimanual motor sequences. *Neuroimage* **19**, 1405-1416 (2003).
10. Debaere, F. *et al*. Brain areas involved in interlimb coordination: a distributed network. *Neuroimage* **14**, 947-958 (2001).
11. Dhamala, M. *et al*. Neural correlates of the complexity of rhythmic finger tapping. *Neuroimage* **20**, 918-926 (2003).
12. Dreher, J. C. & Grafman, J. The roles of the cerebellum and basal ganglia in timing and error prediction. *Eur. J. Neurosci.* **16**, 1609-1619 (2002).
13. Ehrsson, H. H., *et al*. Cortical activity in precision-versus power-grip tasks: an fMRI study. *J. Neurophysiol.* **83**, 528-536 (2000).
14. Ehrsson, H. H., Fagergren, E. & Forssberg, H. Differential fronto-parietal activation depending on force used in a precision grip task: an fMRI study. *J. Neurophysiol.* **85**, 2613-2623 (2001).
15. Ehrsson, H. H., Fagergren, A., Johansson, R. S. & Forssberg, H. Evidence for the involvement of the posterior parietal cortex in coordination of fingertip forces for grasp stability in manipulation. *J. Neurophysiol.* **90**, 2978-2986 (2003).
16. Foltys, H. *et al*. Power grip disinhibits the ipsilateral sensorimotor cortex: a TMS and fMRI study. *Neuroimage* **19**, 332-340 (2003).
17. Hamzei, F. *et al*. Visuomotor control within a distributed parieto-frontal network. *Exp. Brain Res.* **146**, 273-281 (2002).
18. Hamzei, F. *et al*. Reduction of excitability (“inhibition”) in the ipsilateral primary motor cortex is mirrored by fMRI signal decreases. *Neuroimage* **17**, 490-496 (2002).
19. Haslinger, B. *et al*. The role of lateral premotor-cerebellar-parietal circuits in motor sequence control: a parametric fMRI study. *Cogn. Brain Res.* **13**, 159-168 (2002).
20. Indovina, I. & Sanes, J. N. Combined visual attention and finger movement effects on human brain representations. *Exp. Brain Res.* **140**, 265-279 (2001).
21. Indovina, I. & Sanes, J. N. On somatotopic representation centers for finger movements in human primary motor cortex and supplementary motor area. *Neuroimage* **13**, 1027-1034 (2001).
22. Ino, T., Asada, T., Ito, J., Kimura, T. & Fukuyama, H. Parieto-frontal networks for clock drawing revealed with fMRI. *Neurosci. Res.* **45**, 71-77 (2003).
23. Jancke, L., Loose, R., Lutz, K., Specht, K. & Shah, N. J. Cortical activations during paced finger-tapping applying visual and auditory pacing stimuli. *Cogn. Brain Res.* **10**, 51-66 (2000).
24. Jantzen, K. J., Steinberg, F. L. & Kelso, J. A. Practice-dependent modulation of neural activity during human sensorimotor coordination: a functional Magnetic Resonance Imaging study. *Neurosci. Lett.* **332**, 205-209 (2002).
25. Johansen-Berg, H. & Matthews, P. M. Attention to movement modulates activity in sensori-motor areas, including primary motor cortex. *Exp. Brain Res.* **142**, 13-24 (2002).
26. Kawashima, R. *et al*. Human cerebellum plays an important role in memory-timed finger movement: an fMRI study. *J. Neurophysiol.* **83**, 1079-1087 (2000).
27. Kobayashi, M., Hutchinson, S., Schlaug, G. & Pascual-Leone, A. Ipsilateral motor cortex activation on functional magnetic resonance imaging during unilateral hand movements is related to interhemispheric interactions. *Neuroimage* **20**, 2259-2270 (2003).
28. Koski, L. *et al*. Modulation of motor and premotor activity during imitation of target-directed actions. *Cereb. Cortex* **12**, 847-855 (2002).
29. Kuhtz-Buschbeck, J. P., Ehrsson, H. H. & Forssberg, H. Human brain activity in the control of fine static precision grip forces: an fMRI study. *Eur. J. Neurosci.* **14**, 382-390 (2001).
30. Liddle, P. F., Kiehl, K. A. & Smith, A. M. Event-related fMRI study of response inhibition. *Hum. Brain Mapp.* **12**, 100-109 (2001).
31. Lotze, M., Braun, C., Birbaumer, N., Anders, S. & Cohen, L. G. Motor learning elicited by voluntary drive. *Brain* **126**, 866-872 (2003).
32. Lotze, M., Seggewies, G., Erb, M., Grodd, W. & Birbaumer, N. The representation of articulation in the primary sensorimotor cortex. *Neuroreport* **11**, 2985-2989 (2000).
33. Matsuo, K. *et al*. Finger movements lighten neural loads in the recognition of ideographic characters. Brain. Res. *Cogn. Brain Res.* **17**, 263-272 (2003).
34. Mattay, V. S. *et al*. Hemispheric control of motor function: a whole brain echo planar fMRI study. *Psychiatry Res.: Neuroimaging Sect.* **83**, 7-22 (1998).
35. Mayville, J.M., Jantzen, K, J., Fuchs, A., Steinberg, F. L. & Kelso, J. A. Cortical and subcortical networks underlying syncopated and synchronized coordination revealed using fMRI. Functional magnetic resonance imaging. *Hum. Brain Mapp.* **17**, 214-229 (2002).
36. Muller, R. A., Kleinhans, N., Pierce, K., Kemmotsu, N. & Courchesne, E. Functional MRI of motor sequence acquisition: effects of learning stage and performance. *Cogn. Brain Res.* **14**, 277-293 (2002).
37. Rao, S. M. *et al*. Distributed neural systems underlying the timing of movements. *J. Neurosci*. **17**, 5528-5535 (1997).
38. Riecker, A., Wildgruber, D., Mathiak, K., Grodd, W. & Ackermann, H. Parametric analysis of rate-dependent hemodynamic response functions of cortical and subcortical brain structures during auditorily cued finger tapping: a fMRI study. *Neuroimage* **18**, 731-739 (2003).
39. Rotte, M., Kanowski, M. & Heinze, H. J. Functional magnetic resonance imaging for the evaluation of the motor system: primary and secondary brain areas in different motor tasks. *Stereotact. Funct. Neurosurg*. **78**, 3-16 (2002).
40. Rowe, J. *et al*. Attention to action in Parkinson’s disease: impaired effective connectivity among frontal cortical regions. *Brain* **125**, 276-289 (2002).
41. Sakai, K., *et al*. What and when: parallel and convergent processing in motor control. *J. Neurosci.* **20**, 2691-2700 (2000).
42. Schubotz, R. I. & von Cramon, D. Y. Interval and ordinal properties of sequences are associated with distinct premotor areas. *Cereb. Cortex* **11**, 210-222 (2001).
43. Stippich, C., Ochmann, H. & Sartor, K. Somatotopic mapping of the human primary sensorimotor cortex during motor imagery and motor execution by functional magnetic resonance imaging. *Neurosci. Lett.* **331**, 50-54 (2002).
44. Stoeckel, M.C. *et al*. A fronto-parietal circuit for tactile object discrimination: an event-related fMRI study. *Neuroimage* **19**, 1103-1114 (2003).
45. Sugio, T., Ogawa, K. & Inui, T. Neural correlates of semantic effects on grasping familiar objects. *NeuroReport* **14**, 2297-2301 (2003).
46. Toma, K., *et al*. The role of the human supplementary motor area in reactive motor operation. *Neurosci. Lett.* **344**, 177-180 (2003).
47. Toni, I., Krams, M., Turner, R. & Passingham, R. E. The time course of changes during motor sequence learning: a whole-brain fMRI study. *Neuroimage* **8**, 50-61 (1998).
48. Toni, I. *et al*. Multiple movement representations in the human brain: an event-related fMRI study. *J. Cogn. Neurosci.* **14**, 769-784 (2002).
49. Vaillancourt, D. E., Thulborn, K. R. & Corcos, D. M. Neural basis for the processes that underlie visually guided and internally guided force control in humans. *J. Neurophysiol.* **90**, 3330-3340 (2003).
50. Watanabe, J. *et al*. The human prefrontal and parietal association cortices are involved in NO-GO performances: an event-related fMRI study. *Neuroimage* **17**, 1207-1216 (2002).
51. Winterer, G., Adams, C. M., Jones, D. W. & Knutson, B. Volition to action - An event-related fMRI study. *Neuroimage* **17**, 851-858 (2002).
